# Supplementary material for: Formulation and Characterization of β-Cyclodextrins–Nitazoxanide Inclusion Complexes: Enhanced Solubility, In Vitro Drug Release, and Antiviral Activity in Vero Cells
Source: Pharmaceutics. 2024 Nov 21;16(12):1494. doi: 10.3390/pharmaceutics16121494 (PMC11677481; doi:10.3390/pharmaceutics16121494)
Supplement: Supplementary file 1 [file pharmaceutics-16-01494-s001.zip › pharmaceutics-3333848-supplementary.pdf]

Formulation and Characterization of β-Cyclodextrins–Nitazoxanide Inclusion Complexes: Enhanced Solubility, In Vitro Drug Release, and Antiviral Activity in Vero Cells

Kuppu Sakthi Velu [1,†], Sonamuthu Jegatheeswaran [2], Muhammad Saeed Akhtar [3,†], Mohammad Rizwan Khan [4], Sonaimuthu Mohandoss [1,*] and Naushad Ahmad [4,*]

[1] School of Chemical Engineering, Yeungnam University, Gyeongsan 38541, Republic of Korea; sakthi.velu4@yu.ac.kr
[2] BioMe Live Analytical Centre, Karaikudi 630003, Tamil Nadu, India; sjwaran90@gmail.com
[3] Department of Chemistry, Yeungnam University, Gyeongsan 38541, Republic of Korea; passions.malik@gmail.com
[4] Department of Chemistry, College of Science, King Saud University, Riyadh 11451, Saudi Arabia; mrkhan@ksu.edu.sa
* Correspondence: drsmohandoss@yu.ac.kr (S.M.); anaushad@ksu.edu.sa (N.A.)
† These authors contributed equally to this work.

Table S1. UV-Vis spectra of NTX (422 nm) with various concentration of β-CD at pH 7.4.

β-CD concentration	Absorbance	1/[β-CD]	1/[A-A_0]
0	0.257	0	0
0.002	0.397	500	7.142
0.004	0.586	250	3.039
0.006	0.802	166.66	1.834
0.008	0.983	125	1.377
0.010	1.198	100	1.062
0.012	1.338	83.33	0.925
Binding constant (K) (M^{-1})			61.26
Gibbs free energy (ΔG) (Kcal/mol)			-10.36

Table S2. UV-Vis spectra of NTX (422 nm) with various concentration of Hβ-CD at pH 7.4.

Hβ-CD concentration	Absorbance	[1/Hβ-CD]	[1/A-A_0]
0	0.257	0	0
0.002	0.512	500	3.921
0.004	0.737	250	2.083
0.006	0.932	166.66	1.481
0.008	1.155	125	1.113
0.010	1.406	100	0.870
0.012	1.654	83.33	0.715
Binding constant (K) (M^{-1})			94.18
Gibbs free energy (ΔG) (Kcal/mol)			-11.45

Table S3. Molecular docking studies using PatchDock and FireDock servers scores for the top 5 docked models of β-CD:NTX inclusion complex.

	PatchDock server			FireDock server			
S. No.	Score	Area (Å^2)	ACE (kcal/mol)	Global Energy (kcal/mol)	Attractive VdW (kcal/mol)	Repulsive VdW (kcal/mol)	ACE (kcal/mol)
1	3664	441.60	-336.83	-32.16	-12.10	2.63	-11.98
2	3536	420.50	-317.00	-31.21	-12.25	10.43	-13.58
3	3520	463.10	-344.76	-29.47	-10.51	10.28	-13.80
4	3466	419.50	-331.76	-27.73	-12.37	14.18	-13.08
5	3428	410.40	-319.72	-27.73	-11.34	6.10	-10.81

Table S4. Molecular docking studies using PatchDock and FireDock servers scores for the top 5 docked models of Hβ-CD:NTX inclusion complex.

	PatchDock server			FireDock server			
S. No.	Score	Area (Å^2)	ACE (kcal/mol)	Global Energy (kcal/mol)	Attractive VdW (kcal/mol)	Repulsive VdW (kcal/mol)	ACE (kcal/mol)
1	3520	437.60	-342.80	-45.23	-14.22	2.39	-16.40
2	3504	429.60	-282.75	-42.63	-13.56	1.69	-15.20
3	3414	433.10	-337.80	-38.14	-12.21	3.59	-14.30
4	3380	396.40	-317.04	-37.92	-13.55	4.26	-13.83
5	3340	410.30	-317.44	-37.54	-14.62	3.84	-13.18
